# IDeglira vs insulin degludec for type 2 diabetes: a systematic review and meta-analysis

**DOI:** 10.3389/fendo.2025.1643386

**Published:** 2025-09-01

**Authors:** Yang Liu, Xuejing Li, Jie Yang, Suhui Qie, Xiaoli Wang, Xianying Wang, Yingying Zheng

**Affiliations:** Department of Pharmacy, Hebei Medical University Third Hospital, Shijiazhuang, China

**Keywords:** IDegLira, insulin degludec, type 2 diabetes, randomized controlled trials, meta-analysis

## Abstract

**Objectives:**

This systematic review and meta-analysis was to evaluate and compare the efficacy and safety profiles of IDegLira versus insulin degludec in the management of type 2 diabetes (T2D).

**Methods:**

A comprehensive search was systematically conducted across PubMed, Embase, the Cochrane Library, and ClinicalTrials.gov from their inception until March 11, 2025. The search focused on randomized controlled trials (RCTs) that compared IDegLira with insulin degludec in adult patients with T2D. The primary outcomes of interest included change in glycated hemoglobin (HbA1c) and body weight. Random-effects meta-analyses were performed using RevMan 5.4 and Stata 16.0 software.

**Results:**

A total of six eligible RCTs, encompassing 3,393 patients (2,075 receiving IDegLira and 1,318 receiving insulin degludec), were included in the analysis. Treatment with IDegLira resulted in significant reductions in HbA1c (MD -0.79%, 95%CI: -1.03% to -0.54%), body weight (MD -1.62 kg, 95% CI: -2.13 kg to -1.11 kg), fasting plasma glucose (MD -0.45 mmol/L, 95% CI: -0.77 mmol/L to -0.14 mmol/L), self-measured plasma glucose (MD -1.00 mmol/L, 95% CI: -1.42 mmol/L to -0.59 mmol/L), and systolic blood pressure (MD -2.23 mmHg, 95% CI: -3.63 mmHg to -0.82 mmHg). In comparison to insulin degludec, IDegLira demonstrated superior blood glucose control, as evidenced by a higher proportion of patients achieving HbA1c levels below 7.0% and 6.5%, as well as those achieving these targets without weight gain and severe or blood glucose-confirmed hypoglycemic episodes. Additionally, patients treated with IDegLira required significantly lower daily insulin doses. Notably, the risk of severe or blood glucose-confirmed symptomatic hypoglycemia, adverse events, and severe adverse events was comparable between IDegLira and insulin degludec.

**Conclusions:**

This meta-analysis provides compelling evidence that IDegLira offers superior glycemic control and more favorable effects on body weight compared to insulin degludec, while maintaining a comparable safety profile.

## 1 Introduction

Type 2 diabetes (T2D) poses a significant global health challenge, with its prevalence escalating annually. In 2019 alone, 463 million adults were affected, and this number is projected to surge to 700 million by 2045 (1). T2D is marked by progressive β-cell dysfunction and insulin resistance, often necessitating increasingly intensive treatment over time (2). Initially, lifestyle modifications and oral antidiabetic medications serve as the cornerstone of therapy. However, many patients eventually require insulin treatment to effectively manage their blood glucose levels (3). Basal insulin analogs, such as insulin degludec, have gained widespread acceptance due to their extended duration of action and reduced variability in glucose-lowering effects compared to earlier insulin formulations (4). Despite these benefits, insulin therapy is not without challenges. It carries risks of hypoglycemia, weight gain, and the need for careful dose titration, all of which can impact treatment adherence and long-term health outcomes (5). On the other hand, glucagon-like peptide-1 receptor agonists (GLP-1 RAs) have emerged as a valuable addition to the antidiabetic armamentarium. These agents promote glucose-dependent insulin secretion, suppress glucagon release, slow gastric emptying, and enhance satiety, often leading to weight loss rather than gain (6). Their use, however, is primarily limited by gastrointestinal side effects, which are typically dose-dependent and tend to resolve over the course of treatment (7).

IDegLira is a once-daily, subcutaneously administered combination therapy that combines insulin degludec with the GLP-1 RA liraglutide in a fixed ratio (8). This formulation was developed to leverage the complementary mechanisms of action of these two drug classes while potentially mitigating their individual limitations. By integrating a basal insulin with a GLP-1 RA, IDegLira addresses multiple pathophysiological defects in T2D, potentially reducing the required insulin dose and associated side effects. Moreover, the convenience of a single daily injection may enhance patients’ adherence to therapy.

Several global DUAL randomized controlled trials (RCTs) have compared IDegLira with insulin degludec (9–11). However, individual studies may lack sufficient statistical power to detect differences in certain outcomes. Currently, two meta-analyses have compared IDegLira with various antidiabetic drugs to assess its initial efficacy and safety in treating T2D patients (12, 13). Nevertheless, these analyses combined different antidiabetic drugs into a single group without conducting subgroup analyses based on specific drugs. Therefore, we conducted this systematic review and meta-analysis to provide a comprehensive evaluation of the efficacy and safety of IDegLira versus insulin degludec in patients with T2D.

## 2 Methods

The protocol of this meta-analysis has been registered in PROSPERO (CRD420251038517). This systematic review and meta-analysis followed the Preferred Reporting Items for Systematic reviews and Meta-analyses (PRISMA) guidelines for conducting a high-quality meta-analysis (14, 15).

### 2.1 Search strategy and selection criteria

To identify RCTs comparing the efficacy and safety of IDegLira with insulin degludec in patients with T2D, we conducted a comprehensive search across several databases. These included PubMed, Embase, the Cochrane Library, and ClinicalTrials.gov, covering the period from their inception up until March 11, 2025. The search terms utilized encompassed a range of relevant keywords, such as diabetes mellitus, diabetes, T2DM, DM, IDegLira, Xultophy, insulin degludec and liraglutide, degludec/liraglutide, degludec plus liraglutide, degludec, and Tresiba. A detailed search strategy is outlined in **Supplementary Table 1**.

The specific inclusion criteria for this study were as follows: (1) The study design must be a RCT; (2) Participants should be patients with T2D; (3) The intervention is IDegLira; (4) The control group should receive insulin degludec; and (5) The study must report at least one of the following outcomes: change in HbA1c, change in body weight, patients with HbA1c <7%, patients with HbA1c <6.5%, HbA1c <7.0% without weight gain and without severe or blood glucose-confirmed hypoglycemic episodes, HbA1c <6.5% without weight gain and without severe or blood glucose-confirmed hypoglycemic episodes, change in fasting plasma glucose (FPG), change in self-measured plasma glucose (SMPG), change in systolic blood pressure, change in diastolic blood pressure, total daily insulin dose, severe or blood glucose-confirmed symptomatic hypoglycemia, as well as adverse events (AEs) and serious adverse events (SAEs).

Exclusion criteria for this study encompassed reviews, conference abstracts, case reports, meta-analyses, comments, non-English or non-Chinese language publications, RCTs with inadequate or unusable data, and studies with a treatment duration of less than 12 weeks. Two authors independently conducted a literature search based on the predefined inclusion criteria.

### 2.2 Data extraction and quality assessment

The extracted data encompassed a comprehensive set of items, including baseline characteristics (such as trial name, country, study arms, number of patients, age, male ratio, body weight, body-mass index, HbA1c levels, diabetes duration, and treatment duration) and the outcomes of interest (which included change in HbA1c, change in body weight, patients with HbA1c <7%, patients with HbA1c <6.5%, HbA1c <7.0% without weight gain and without severe or blood glucose-confirmed hypoglycemic episodes, HbA1c <6.5% without weight gain and without severe or blood glucose-confirmed hypoglycemic episodes, change in FPG, change in SMPG, change in systolic blood pressure, change in diastolic blood pressure, total daily insulin dose, severe or blood glucose-confirmed symptomatic hypoglycemia, as well as AEs and SAEs. Our primary outcomes were change in HbA1c and body weight. Two investigators independently extracted the data using a standardized, pre-formatted Excel template, with a third investigator serving as an arbiter in case of discrepancies. For continuous variables, the mean and standard deviation were estimated using the methodologies proposed by Hozo et al. and Wan et al. (16, 17). Because of some participants withdrew during clinical trials, almost all outcome-specific analyses included fewer participants than the total study population and the most withdrawn patients were for adverse events and/or met withdrawal criteria. All data were extracted directly from publications and authors of these included studies were not contacted for additional information.

The risk of bias within individual studies was evaluated utilizing the Cochrane Risk of Bias Tool (18), which included seven domains: random sequence generation, allocation concealment, blinding of participants and personnel, blinding of outcome assessment, incomplete outcome data, selective reporting, and other bias. Each domain was assessed and categorized as having a “low risk,” “high risk,” or “unclear risk” of bias.

### 2.3 Statistical analysis

We performed meta-analyses when at least two studies provided relevant outcome data. For continuous outcomes, we computed the mean difference (MD), while for dichotomous data, we calculated the risk ratio (RR). All effect estimates were presented alongside 95% confidence intervals (CI). The heterogeneity among studies was assessed using Cochrane’s Q statistic and the I² statistic. Pooled analyses were conducted employing a random-effects model to account for potential variability. We evaluated publication bias through a visual examination of funnel plots. To identify the sources of heterogeneity, we carried out subgroup analysis and meta-regression analysis. Furthermore, the robustness of our meta-analysis models was rigorously tested using sensitivity analyses based on the leave-one-out method. All statistical procedures were executed using RevMan 5.4 software and Stata 16.0 software.

## 3 Results

### 3.1 Search results and quality assessment

**Figure 1:**
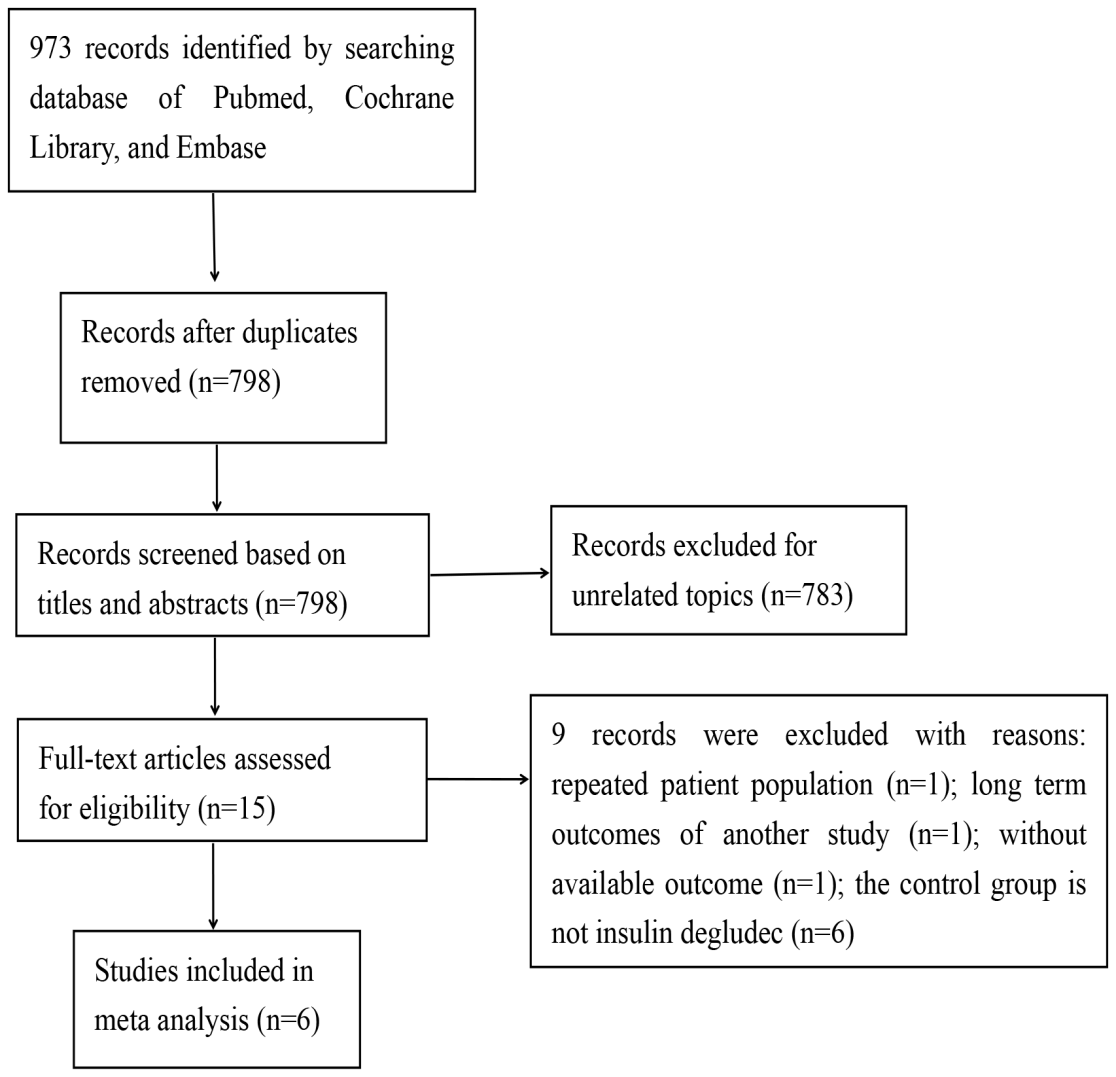
The selection process of included studies.

A comprehensive search across four databases yielded a total of 973 records. After eliminating duplicate entries, 798 records were retained for preliminary screening based on titles and abstracts. Of these, 783 references were excluded during this initial phase, leaving 15 studies for more in-depth evaluation. Ultimately, 6 records met the eligibility criteria and were included in this meta-analysis, while 9 records were excluded (**Figure 1**).

**Figure 2:**
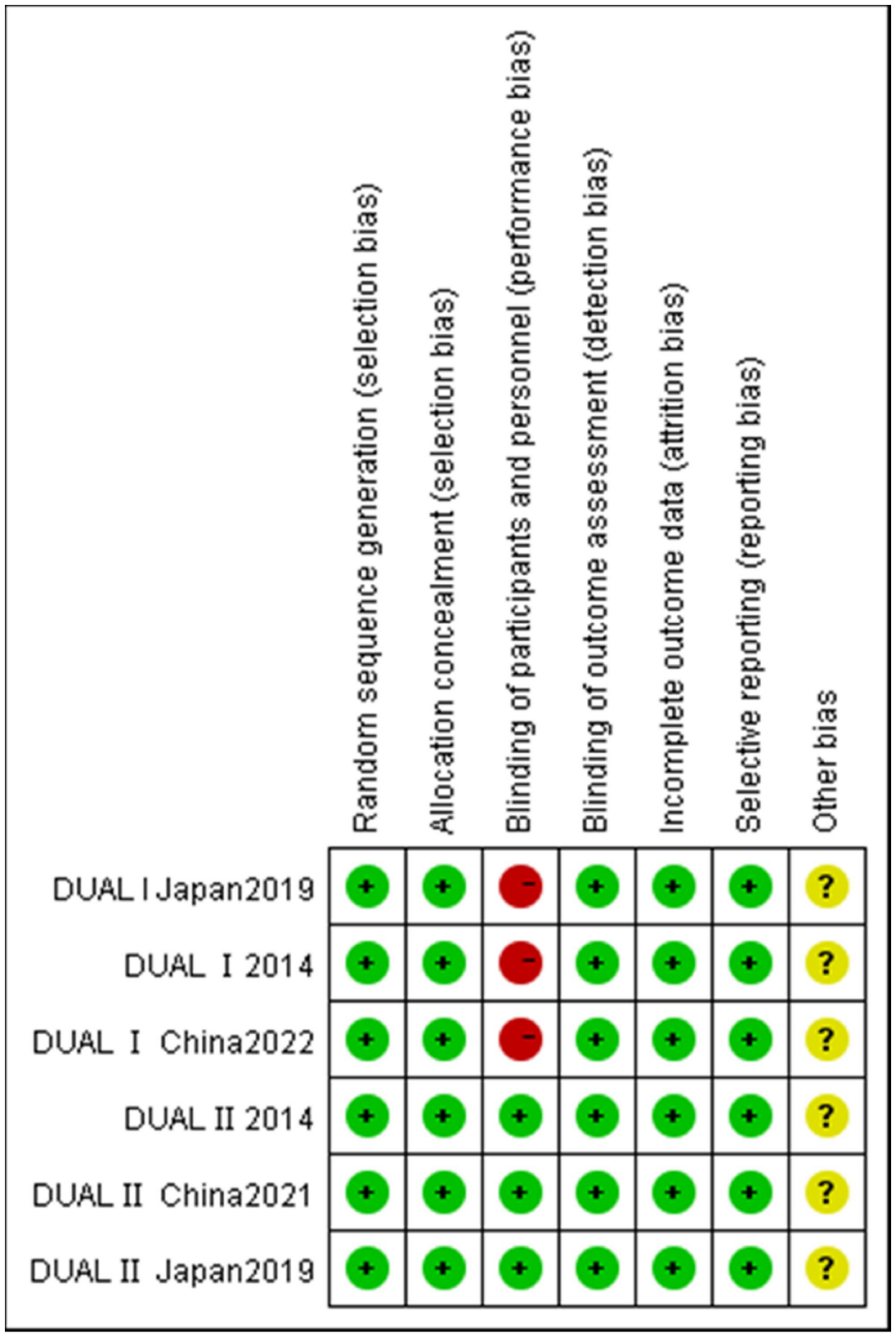
Quality assessment of included studies.

The characteristics of the trials incorporated in this analysis are summarized in **Table 1**. This meta-analysis encompasses 6 RCTs, involving a cumulative sample of 3,393 individuals (7, 9–11, 19, 20). Geographically, two of the included studies were conducted in China, two in Japan, and the remaining two were global or multinational in scope. The duration of these studies ranged from 26 to 52 weeks. Baseline characteristics of the participants varied across studies, with mean ages ranging from 54.5 to 58 years and initial HbA1c levels spanning from 8.2% to 8.96%. Applying the Cochrane criteria, the overall risk of bias across all studies was assessed as low. The risk of bias assessment is depicted in **Figure 2**.

**Table 1:** Characteristics of the included studies and patients.

Trial name	Country	Study arms	Co-medications	Patients	Age (years)	Male, n (%)	Body weight (kg)	Body-mass index (kg/m^2^)	HbA1c (%)	Duration of diabetes (years)	Treatment duration (weeks)
DUAL I China2022 (6)	China	IDegLira	Metformin ± one other oral antidiabetic drugs	361	54.5 ± 10.3	219 (60.7)	74.8 ± 14.3	27.0 ± 3.9	8.20 ± 0.83	8.00 ± 5.33	26
		Degludec		179	55.7 ± 10.2	100 (55.9)	73.2 ± 13.2	26.5 ± 3.6	8.31 ± 0.84	8.63 ± 5.55	
DUAL I Japan2019 (17)	Japan	IDegLira	α-glucosidase inhibitors; thiazolidinediones; sodium-glucose co-transporter-2 inhibitors; glinides; metformin; or sulphonylureas	275	56.9 ± 10.2	194 (70.5)	70.7 ± 12.4	26.1 ± 3.7	8.5 ± 1.1	9.2 ± 6.2	52
		Degludec		271	57.8 ± 9.9	195 (72.0)	72.6 ± 14.5	26.6 ± 4.8	8.5 ± 1.1	9.7 ± 6.0	
DUAL I2014 (4)	UK, USA, Canada, et al	IDegLira	Metformin ± pioglitazone	833	55.1 ± 9.9	435 (52.2)	87.2 ± 19.0	31.2 ± 5.2	8.3 ± 0.9	6.6 ± 5.1	26
		Degludec		413	54.9 ± 9.7	200 (48.4)	87.4 ± 19.2	31.2 ± 5.3	8.3 ± 1.0	7.0 ± 5.3	
DUAL II China2021 (7)	China	IDegLira	Metformin ± one other oral antidiabetic drugs	302	54.5 ± 9.8	183 (60.6)	76.8 ± 13.0	27.5 ± 3.3	8.93 ± 1.20	11.52 ± 5.9	26
		Degludec		151	55.3 ± 10.0	91 (60.3)	74.3 ± 11.4	27.0 ± 2.9	8.96 ± 1.17	11.33 ± 6.3	
DUAL II Japan2019 (8)	Japan	IDegLira	Metformin ± one other oral antidiabetic drugs	105	56.6 ± 10.4	70 (66.7)	73.9 ± 11.9	27.3 ± 3.1	8.61 ± 0.88	14.33 ± 7.79	26
		Degludec		105	55.5 ± 10.0	63 (60)	75.5 ± 14.0	28.1 ± 4.4	8.56 ± 0.80	13.77 ± 7.46	
DUAL II2014 (16)	Denmark, Bulgaria, Switzerland, et al	IDegLira	Metformin ± sulfonylurea/glinides	199	57 ± 9	111 (56)	95.4 ± 19	33.6 ± 6	8.7 ± 0.7	10 ± 6	26
		Degludec		199	58 ± 11	105 (53)	93.5 ± 20	33.8 ± 6	8.8 ± 0.7	11 ± 7	

### 3.2 Efficacy outcomes

**Figure 3:**
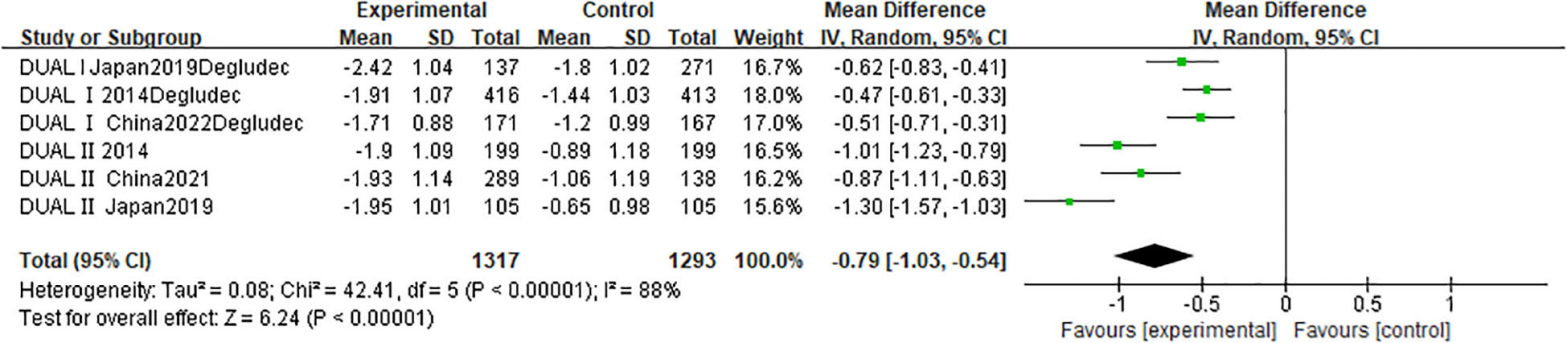
Forest plot for change in HbA1c.

A pooled analysis of six RCTs (n = 2,610) evaluated change in HbA1c following IDegLira versus insulin degludec treatment (**Figure 3**). Results showed a statistically significant HbA1c reduction favoring IDegLira (MD -0.79%; 95% CI -1.03 to -0.54; p < 0.00001), though substantial heterogeneity existed (I² = 88%; p < 0.00001). Funnel plot analysis (**Supplementary Figure S1**) indicated no publication bias. Similarly, change in body weight (six studies; n = 2,610) was significantly reduced with IDegLira versus insulin degludec (MD -1.62 kg; 95% CI -2.13 to -1.11; p < 0.00001), with moderate heterogeneity (I² = 74%; p = 0.002) and no bias per funnel plot (**Supplementary Figure S3**).

IDegLira also improved glycemic targets: HbA1c <7.0% (six studies; n = 2,610): RR 1.93 (95% CI 1.45–2.56; p < 0.00001; I² = 94%) (**Supplementary Figure S4**); HbA1c <6.5% (six studies; n = 2,610): RR 2.51 (95% CI 1.77–3.55; p < 0.00001; I² = 91%) (**Supplementary Figure S5**). Funnel plots (**Supplementary Figures S6, S7**) showed no publication bias.

Composite outcomes further favored IDegLira: HbA1c <7.0% without weight gain and without severe or blood glucose-confirmed hypoglycemia (six studies; n = 2,636): RR 3.29 (95% CI 2.18–4.96; p < 0.00001; I² = 77%) (**Supplementary Figure S8**); HbA1c <6.5% without weight gain and without severe or blood glucose-confirmed hypoglycemia (six studies; n = 2,610): RR 4.15 (95% CI 2.60–6.62; p < 0.00001; I² = 70%) (**Supplementary Figure S9**). No publication bias was detected (**Supplementary Figures S10, S11**).

IDegLira reduced FPG levels (five studies; n = 1,782) more than insulin degludec (MD -0.45 mmol/L; 95% CI -0.77 to -0.14; p = 0.005; I² = 30%) (**Supplementary Figure S12**). Greater reductions in SMPG (four studies; n = 1,348) with IDegLira (MD -1.00 mmol/L; 95% CI -1.42 to -0.59; p < 0.00001; I² = 54%) (**Supplementary Figure S13**). Funnel plots (**Supplementary Figures S14, S15**) showed symmetry.

Significantly lower systolic blood pressure with IDegLira than with insulin degludec (MD -2.23 mmHg; 95% CI -3.63 to -0.82; p = 0.002; I² = 0%) (**Supplementary Figure S16**). No between-group difference was found in diastolic blood pressure (MD 0.17 mmHg; 95% CI -0.74 to 1.08; p = 0.72; I² = 0%) (**Supplementary Figure S17**). Daily insulin dose was lower with IDegLira than with insulin degludec (MD -6.83 U; 95% CI -11.07 to -2.60; p = 0.002; I² = 91%) (**Supplementary Figure S18**). No publication bias was evident (**Supplementary Figures S19–S21**).

### 3.3 Safety outcomes

Pooled safety data demonstrated comparable AE and SAE rates between groups (**Supplementary Figures S22-S23**). However, IDegLira was associated with significantly less severe/blood glucose-confirmed hypoglycemia (**Supplementary Figure S24**). Symmetrical funnel plots (**Supplementary Figures S25-S27**) indicated no publication bias for safety endpoints.

### 3.4 Meta-regression

As summarized in **Table 2**, baseline characteristics-including age, male, and baseline HbA1c-showed no significant association with any of these outcomes (p > 0.05). However, diabetes duration significantly contributed to heterogeneity in both change in HbA1c and patients with HbA1c <7%. Body-mass index was a significant modifier of heterogeneity for change in body. Notably, neither BMI nor diabetes duration influenced heterogeneity in the remaining outcomes (p > 0.05).

**Table 2:** Meta-regression on factors affecting change in HbA1c, change in body weight, and patients with HbA1c < 7%.

Outcome	Independent variable	Coefficient	95% CI		P-value
Change in HbA1c	Age (years)	-0.0861948	-0.4247051	0.2523154	0.519
	Male (%)	-0.6998649	-6.853297	5.453567	0.768
	Body-mass index (kg/m^2^)	-0.0136561	-0.1646452	0.1373329	0.814
	HbA1c (%)	-0.7694006	-2.03982	0.5010189	0.168
	Duration of diabetes (years)	-0.1141489	-0.1707617	-0.0575361	0.005
Change in body weight	Age (years)	-0.2171879	-0.8708044	0.4364286	0.408
	Male (%)	6.234543	-3.022146	15.49123	0.135
	Body-mass index (kg/m^2^)	-0.2206444	-0.3610795	-0.0802094	0.012
	HbA1c (%)	-0.2412306	-3.51932	3.036858	0.848
	Duration of diabetes (years)	0.0442017	-0.3024706	0.390874	0.741
Patients with HbA1c < 7%	Age (years)	-0.0161842	-1.050846	1.018477	0.967
	Male (%)	0.1688957	-17.81999	18.15778	0.980
	Body-mass index (kg/m^2^)	0.0152353	-0.4225448	0.4530155	0.928
	HbA1c (%)	2.69445	-0.3573933	5.746293	0.070
	Duration of diabetes (years)	0.3216948	0.0885241	0.5548655	0.019

### 3.5 Subgroup analysis and sensitivity analysis

Subgroup analyses by race aligned with primary results (**Table 3**). Sensitivity analyses (leave-one-out method; **Supplementary Figures S28–S30**) confirmed robust findings for change in HbA1c, change in body weight, and patients with HbA1c <7%.

**Table 3:** Summary of subgroup analyses for T2DM patients.

Outcomes	Subgroup	Included trials	Included patients	Statistical method	RR/MD (95%CI)
Change in HbA1c	East Asian	4	1383	MD (M-H, Random, 95% CI)	-0.82 [-1.14, -0.49]
	Non-East Asian	2	1227	MD (M-H, Random, 95% CI)	-0.73 [-1.26, -0.20]
Change in body weight	East Asian	4	1383	MD (M-H, Random, 95% CI)	-1.20 [-1.52, -0.87]
	Non-East Asian	2	1227	MD (M-H, Random, 95% CI)	-2.35 [-2.93, -1.77]
Patients with HbA1c < 7%	East Asian	4	1383	RR (M-H, Random, 95% CI)	2.08 [1.29, 3.33]
	Non-East Asian	2	1227	RR (M-H, Random, 95% CI)	1.78 [0.81, 3.92]
Patients with HbA1c < 6.5%	East Asian	4	1383	RR (M-H, Random, 95% CI)	2.89 [1.61, 5.21]
	Non-East Asian	2	1227	RR (M-H, Random, 95% CI)	2.21 [0.92, 5.32]
HbA1c <7.0% without weight gain and without severe or blood glucose-confirmed hypoglycemic episodes	East Asian	4	1409	RR (M-H, Random, 95% CI)	3.38 [1.66, 6.85]
	Non-East Asian	2	1227	RR (M-H, Random, 95% CI)	3.34 [1.82, 6.13]
HbA1c <6.5% without weight gain and without severe or blood glucose-confirmed hypoglycemic episodes	East Asian	4	1409	RR (M-H, Random, 95% CI)	4.28 [1.89, 9.68]
	Non-East Asian	2	1227	RR (M-H, Random, 95% CI)	4.47 [2.44, 8.17]
Change in FPG	East Asian	4	1384	MD (M-H, Random, 95% CI)	-0.33 [-0.61, -0.04]
	Non-East Asian	1	398	MD (M-H, Random, 95% CI)	-0.90 [-1.51, -0.29]
Change in SMPG	East Asian	4	1348	MD (M-H, Random, 95% CI)	-1.00 [-1.42, -0.59]
	Non-East Asian	0	0	MD (M-H, Random, 95% CI)	–
Change in systolic blood pressure	East Asian	4	1383	MD (M-H, Random, 95% CI)	-2.23 [-3.63, -0.82]
	Non-East Asian	0	0	MD (M-H, Random, 95% CI)	–
Change in diastolic blood pressure	East Asian	4	1383	MD (M-H, Random, 95% CI)	0.17 [-0.74, 1.08]
	Non-East Asian	0	0	MD (M-H, Random, 95% CI)	–
Total daily insulin dose	East Asian	4	1692	MD (M-H, Random, 95% CI)	-4.56 [-6.13, -2.99]
	Non-East Asian	1	1224	MD (M-H, Random, 95% CI)	-15.00 [-17.86, -12.14]
Severe or blood glucose-confirmed symptomatic hypoglycaemia	East Asian	4	1424	RR (M-H, Random, 95% CI)	0.73 [0.55, 0.96]
	Non-East Asian	1	824	RR (M-H, Random, 95% CI)	0.82 [0.68, 0.99]
AEs	East Asian	4	1424	RR (M-H, Random, 95% CI)	1.04 [0.98, 1.10]
	Non-East Asian	2	1222	RR (M-H, Random, 95% CI)	1.01 [0.92, 1.12]
SAEs	East Asian	4	1424	RR (M-H, Random, 95% CI)	1.01 [0.61, 1.66]
	Non-East Asian	2	1222	RR (M-H, Random, 95% CI)	0.79 [0.48, 1.31]

## 4 Discussion

This meta-analysis demonstrates IDegLira’s superiority over insulin degludec in terms of lowering blood glucose levels, controlling weight, and reducing insulin dosage. Furthermore, IDegLira showed a significantly lower incidence of severe or blood glucose-confirmed hypoglycemia, while maintaining comparable rates of other adverse events (AEs) and serious adverse events (SAEs). The robustness of these findings was confirmed through subgroup and sensitivity analyses.

Similar background glucose-lowering therapies (e.g., metformin, pioglitazone, and sulphonylureas) were permitted across all included RCTs, with metformin—recommended as first-line therapy by both the American Diabetes Association (ADA) and Chinese Diabetes Society (CDS) guidelines (21, 22)—being the most frequently used. The concurrent use of these agents may have contributed to the observed treatment effects, suggesting that the absolute benefit of the target drug in treatment-naïve populations could be lower than the pooled estimate reported here. Although randomization ensured balanced co-medication distribution between study arms, residual variations in background regimens may explain part of the heterogeneity observed in certain outcomes. Importantly, our primary analysis did not adjust for concomitant medications, which may limit the generalizability of findings to real-world settings where background therapies differ.

Clinical inertia in T2D management is often compounded by the complexity of adding medications to existing polypharmacy regimens (23). Given the established inverse relationship between regimen complexity and patient adherence (24), simplified approaches may help mitigate this inertia. IDegLira offers such simplification: despite both interventions requiring once-daily injections, it eliminates the need for separate insulin and glucagon-like peptide-1 receptor agonist administrations. Notably, its single-dose regimen shows particular promise for deprescribing in elderly patients, improving health outcomes and quality of life (25).

Balancing efficacy and cost is crucial in T2D management due to its substantial financial burden. Pharmacoeconomic analyses are therefore vital for optimizing resource allocation. In the Chinese context, Weng et al. (26) demonstrated that IDegLira is a cost-effective alternative to basal-bolus therapy or glucagon-like peptide-1 receptor agonist monotherapy. Collectively, evidence supports IDegLira as a highly cost-effective intensification strategy for patients uncontrolled on basal insulin.

IDegLira also addresses two key barriers to insulin initiation: weight gain and hypoglycemia (5). Its liraglutide component drove significant weight reduction versus insulin degludec. Additionally, the observed trend towards lower hypoglycemia incidence aligns with global DUAL trial data (27, 28). In summary, IDegLira demonstrates greatest clinical utility as an escalation therapy for patients with type 2 diabetes inadequately controlled on oral agents who require robust HbA1c reduction without weight gain or hypoglycemia penalty, particularly when treatment adherence is a concern.

However, several limitations of our current meta-analysis should be acknowledged. Firstly, the included studies had relatively short follow-up periods, with the longest being only 52 weeks. Consequently, the long-term prognosis for patients receiving IDegLira versus insulin degludec remains uncertain and warrants continued follow-up. Secondly, we observed high heterogeneity in some of the results, which could not be fully explored through subgroup or sensitivity analyses, despite the use of random effects models. Moreover, the lack of blinding in certain studies may have influenced the reporting of specific safety outcomes, such as hypoglycemia. Finally, we do not compared IDegLira with basal insulin with premix insulin or basal bolus regimens for limited number of RCTs.

## 5 Conclusions

Compared with insulin degludec, treatment with IDegLira was superior to insulin degludec in terms of change in HbA1c, change in body weight, patients with HbA1c <7.0%, patients with HbA1c <6.5%, HbA1c <7.0% without weight gain and without severe or blood glucose-confirmed hypoglycemic episodes, HbA1c <6.5% without weight gain and without severe or blood glucose-confirmed hypoglycemic episodes, change in fasting plasma glucose, change in self-measured plasma glucose, and total daily insulin dose, while maintaining a comparable safety profile. This meta-analysis provided evidence of efficacy and safety differences of IDegLira and insulin degludec in T2D patients to guide the clinical practice.

## Data Availability

The original contributions presented in the study are included in the article/**Supplementary Material**. Further inquiries can be directed to the corresponding author.
