# Supplementary material for: IDeglira vs insulin degludec for type 2 diabetes: a systematic review and meta-analysis
Source: Front Endocrinol (Lausanne). 2025 Sep 1;16:1643386. doi: 10.3389/fendo.2025.1643386 (PMC12434752; doi:10.3389/fendo.2025.1643386)
Supplement: Supplementary file 1 [file DataSheet1.doc]

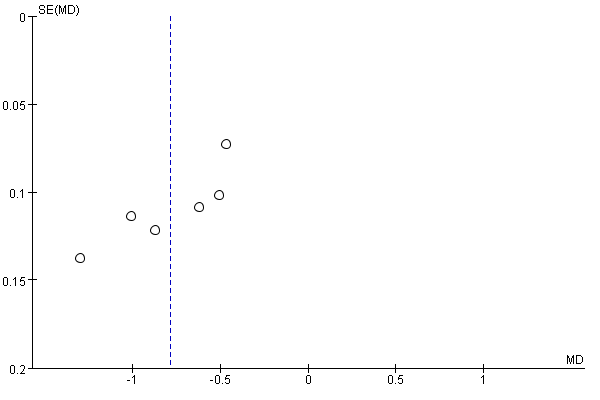


**Supplementary Figure 1 The funnel plot for change in HbA1c**


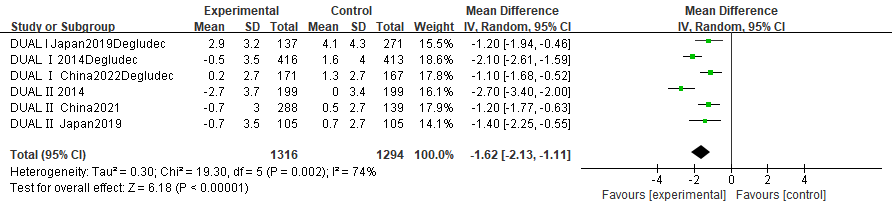


**Supplementary Figure 2 Forest plot for change in body weight**


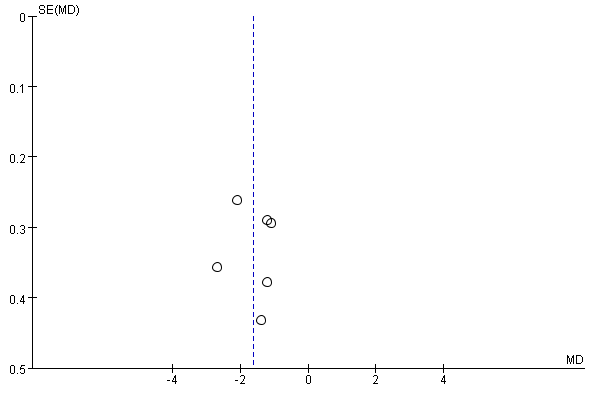


**Supplementary Figure 3 The funnel plot for change in body weight**


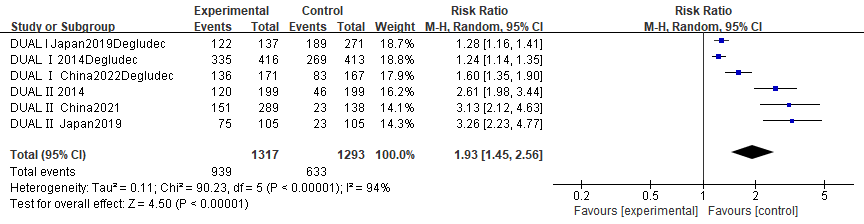


**Supplementary Figure 4 Forest plot for patients with HbA1c < 7%**


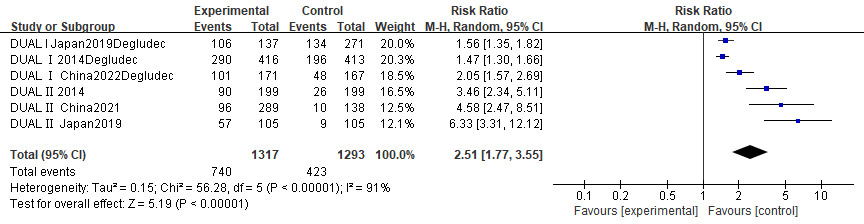


**Supplementary Figure 5 Forest plot for patients with HbA1c < 6.5%**


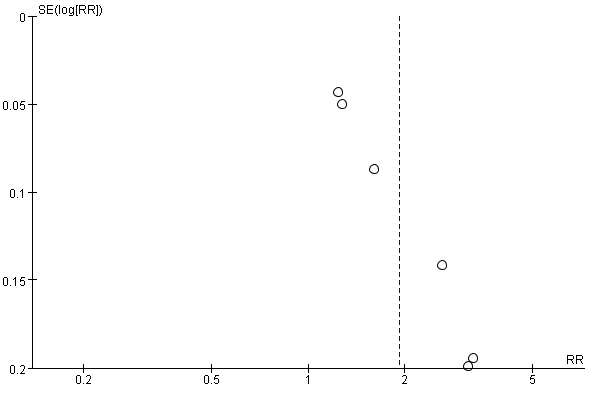


**Supplementary Figure 6 The funnel plot for patients with HbA1c < 7%**


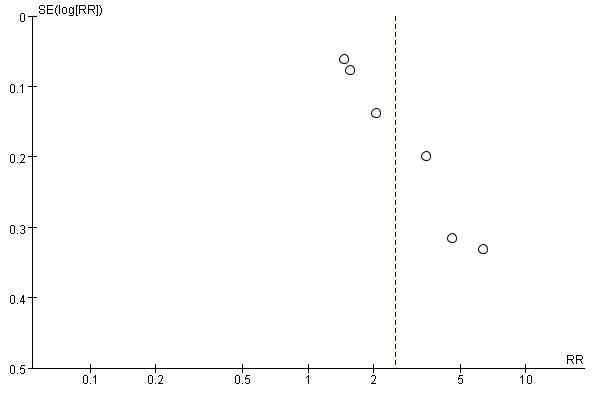


**Supplementary Figure 7 The funnel plot for patients with HbA1c < 6.5%**


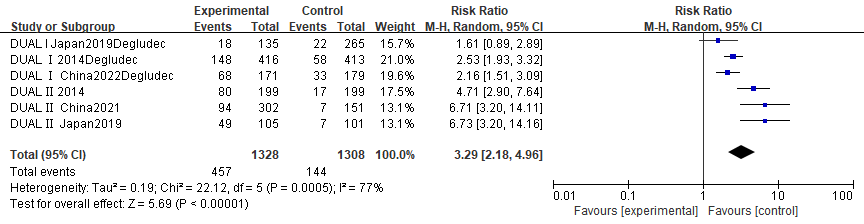


**Supplementary Figure 8 Forest plot for HbA1c <7.0% without weight gain and without severe or BG-confirmed hypoglycemic episodes**


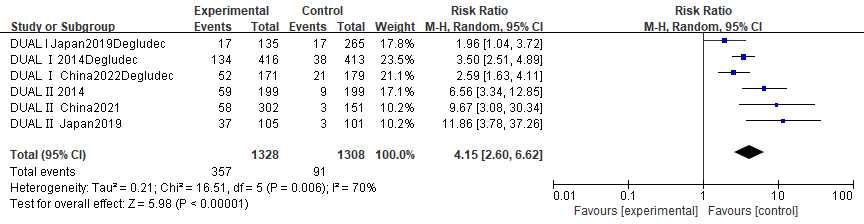


**Supplementary Figure 9 Forest plot for HbA1c <6.5% without weight gain and without severe or BG-confirmed hypoglycemic episodes**


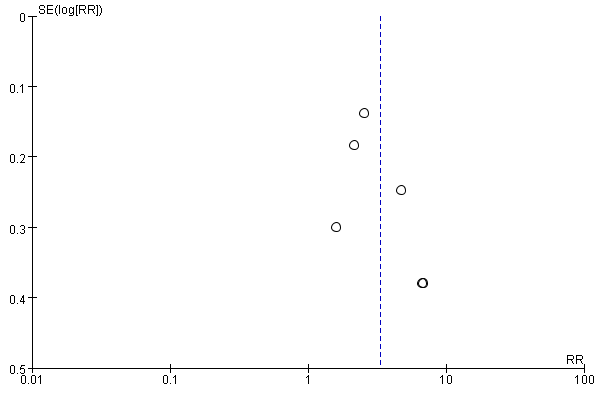


**Supplementary Figure 10 The funnel plot for HbA1c <7.0% without weight gain and without severe or BG-confirmed hypoglycemic episodes**


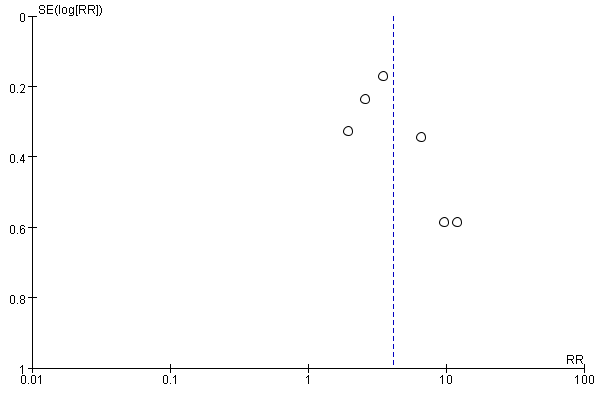


**Supplementary Figure 11 The funnel plot for HbA1c <6.5% without weight gain and without severe or BG-confirmed hypoglycemic episodes**


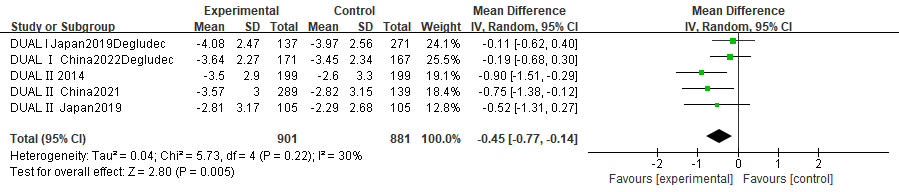


**Supplementary Figure 12 Forest plot for change in FPG**


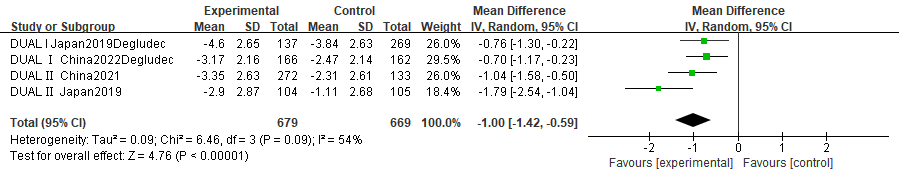


**Supplementary Figure 13 Forest plot for change in SMPG**


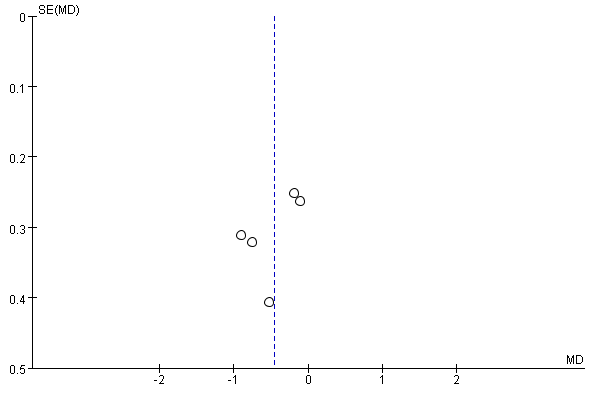


**Supplementary Figure 14 The funnel plot for change in FPG**


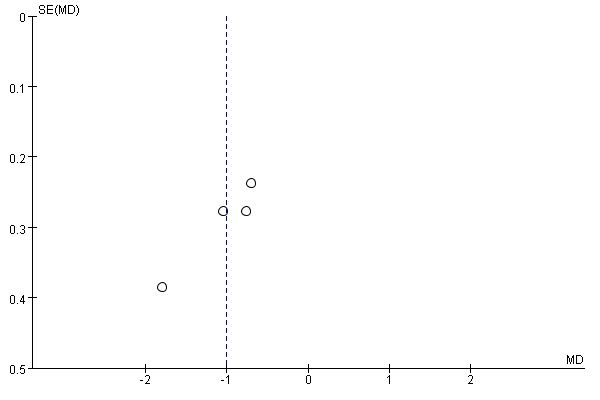


**Supplementary Figure 15 The funnel plot for change in SMPG**


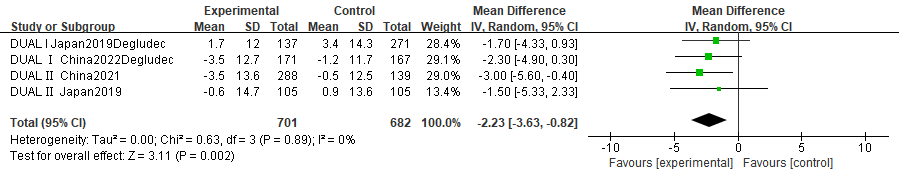


**Supplementary Figure 16 Forest plot for change in SBP**


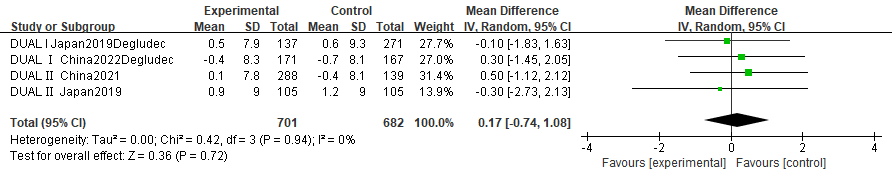


**Supplementary Figure 17 Forest plot for change in DBP**


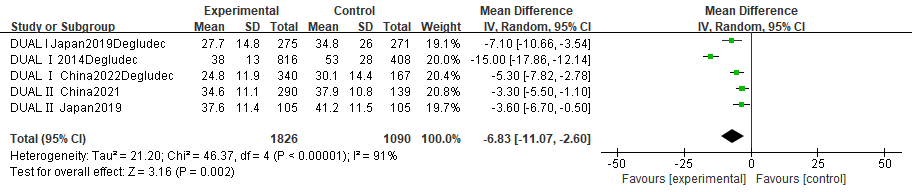


**Supplementary Figure 18 Forest plot for total daily insulin dose**


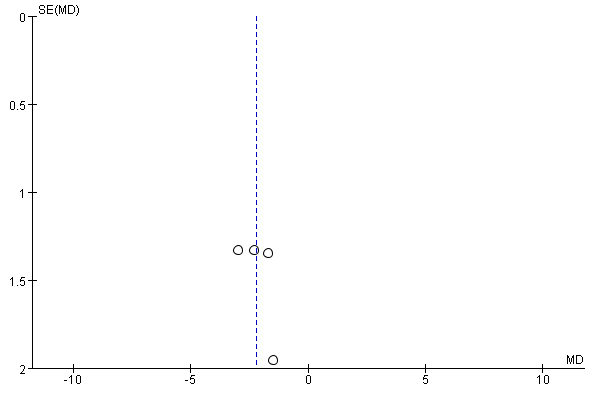


**Supplementary Figure 19 The funnel plot for change in SBP**


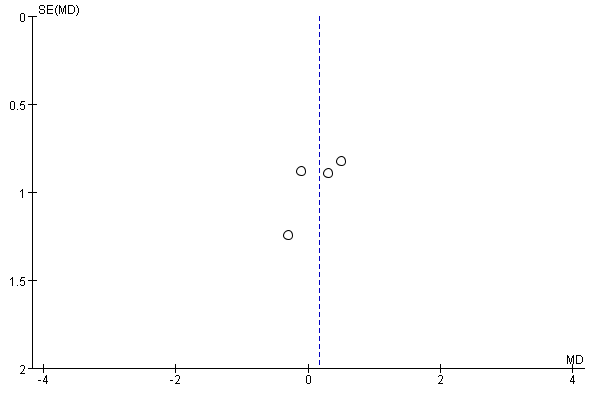


**Supplementary Figure 20 The funnel plot for change in DBP**


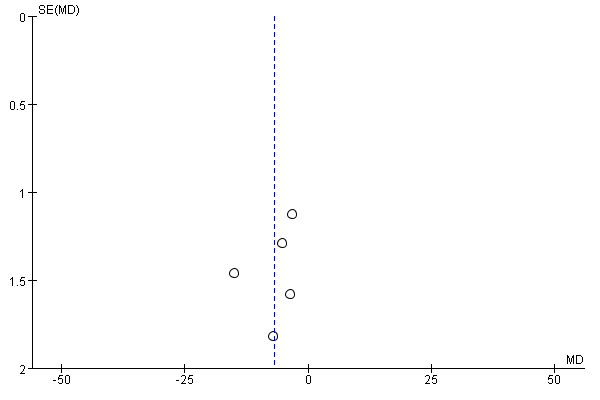


**Supplementary Figure 21 The funnel plot for total daily insulin dose**


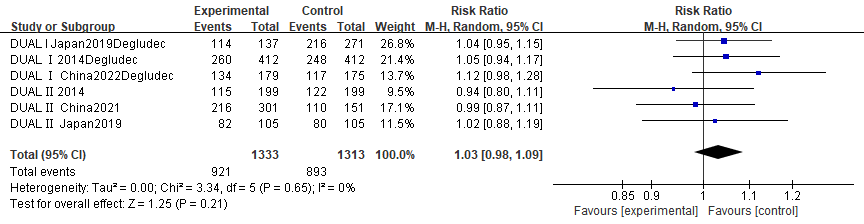


**Supplementary Figure 22 Forest plot for AEs**


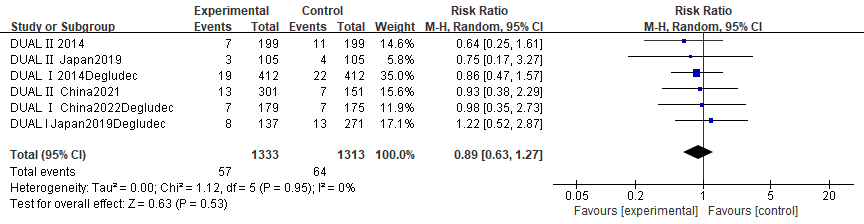


**Supplementary Figure 23 Forest plot for SAEs**


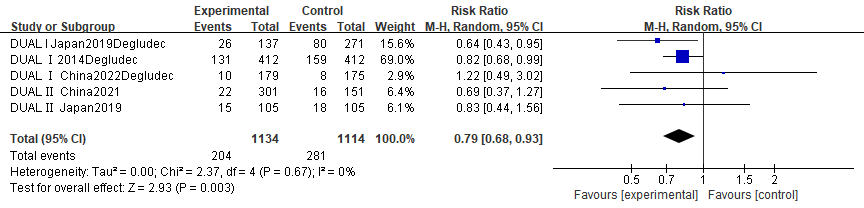


**Supplementary Figure 24 Forest plot for severe or BG-confirmed symptomatic hypoglycaemia**


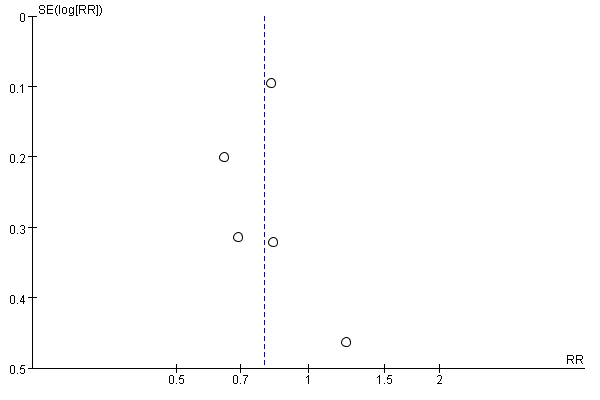


**Supplementary Figure 25 The funnel plot for severe or BG-confirmed symptomatic hypoglycaemia**


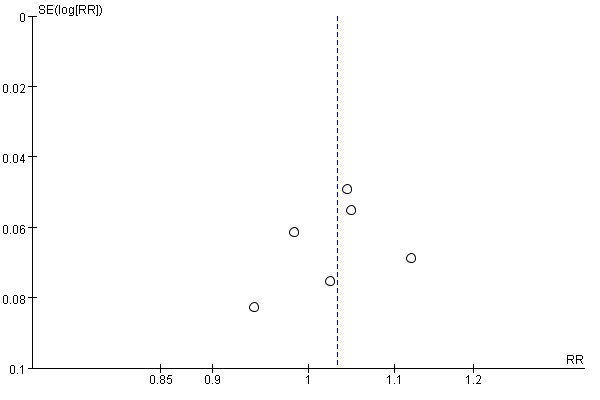


**Supplementary Figure 26 The funnel plot for AEs**


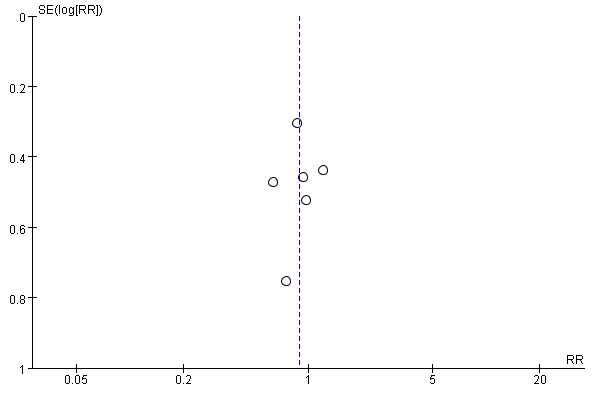


**Supplementary Figure 27 The funnel plot for SAEs**

**Supplementary Figure 28 Sensitivity analysis of change in HbA1c**

**Supplementary Figure 29 Sensitivity analysis of change in body weight**

**Supplementary Figure 30 Sensitivity analysis of patients with HbA1c < 7%**

| **#1** | **diabetes mellitus [Mesh]** |
| --- | --- |
| **#2** | **diabetes [Title]** |
| **#3** | **T2DM [Title]** |
| **#4** | **DM [Title]** |
| **#5** | **#1 OR #2 OR #3 OR #4** |
| **#6** | **IDegLira [Supplementary Concept]** |
| **#7** | **IDegLira [Title]** |
| **#8** | **Xultophy [Title]** |
| **#9** | **Insulin degludec and liraglutide [Title]** |
| **#10** | **degludec/liraglutide [Title]** |
| **#11** | **degludec plus liraglutide** |
| **#12** | **#6 OR #7 OR #8 OR #9 OR #10 OR #11** |
| **#13** | **degludec [Mesh]** |
| **#14** | **Tresiba [Title]** |
| **#15** | **degludec [Title]** |
| **#16** | **#13 OR #14 OR #15** |
| **#17** | **#5 AND #12 AND #16** |

**Supplementary Table1 Search strategies**
